# Functional profiles of coronal and dentin caries in children

**DOI:** 10.1080/20002297.2018.1495976

**Published:** 2018-07-16

**Authors:** Christine A Kressirer, Tsute Chen, Kristie Lake Harriman, Jorge Frias-Lopez, Floyd E Dewhirst, Mary A Tavares, Anne CR Tanner

**Affiliations:** a The Forsyth Institute, Cambridge, USA; b School of Dental Medicine, Harvard University, Boston, USA; c Waterville Clinic, Community Dental Center, Waterville, USA; d Department of Oral Biology, University of Florida, Gainesville, USA

**Keywords:** Metatranscriptome, microbiota, dental plaque, caries, children

## Abstract

**Background**: Dental caries results from a dysbiosis of tooth-associated biofilms and frequently extends through enamel into dentin which has a different structure and composition.

**Objective**: To evaluate the metatranscriptome of caries to determine the metabolic potential of caries communities compared with health.

**Design**: Samples from children, caries-free (CF: *n* = 4) or with coronal (CC: *n* = 5) or dentin (DC: *n* = 5) caries were examined for gene expression potential. Functional profiling was performed using HUMAnN2 (HMP Unified Metabolic Analysis Network).

**Results**: There was increased gene expression diversity in DC compared with CC and CF. Genes in CF included alcohol dehydrogenase from *Neisseria sicca*, methylenetetrahydrofolate reductase from *Streptococcus sanguinis* and choline kinase from streptococci. Genes in CC mapped mainly to *Streptococcus mutans*. Arginine deiminase in DC mapped to *S. sanguinis* and *Actinomyces naeslundii*. Glycerol kinase genes mapped to *S. sanguinis* in all groups whereas glycerol kinase in DC were from *Rothia, Prevotella* and streptococci. Uracil-DNA glycosylase in DC mapped to *Prevotella denticola* and *Actinomyces*. Repressor LexA in DC mapped to *Scardovia wiggsiae, Dialister invisus* and *Veillonella parvula*.

**Conclusions**: Functional profiling revealed enzyme activities in both caries and caries-free communities and clarified marked differences between coronal and dentin caries in bacterial composition and potential gene expression.

## Introduction

Dental caries in children persists as a significant dental and public health challenge in selected, mainly lower income communities [1,2]. Caries results from an imbalance in the oral microbiota with increased acid production from bacterial metabolism after intake of dietary carbohydrates [3]. This increased acid production alters the composition of the microbiome with the suppression of acid-sensitive bacteria [4,5] which changes the bacterial community’s ability to counterbalance the lower local pH in active caries. This pH buffering of the healthy microbiome has been found from bacterial ammonia production from arginine deiminase (ADS) and urease activities [6–9].

The low pH at the tooth surface leads to enamel demineralization, then enamel cavities which can progress into the tooth dentin. The composition of enamel differs from that of dentin, and caries progression in the less mineralized dentin is not associated with as low a pH as observed for enamel caries [10]. Several highly acidogenic and acid-tolerant bacteria have been associated with caries, including *Streptococcus mutans* and *Streptococcus sobrinus* and certain *Lactobacillus, Bifidobacterium, Scardovia* and *Actinomyces* [5]. The microbiota of dentin caries differs from that of enamel caries and includes *Lactobacillus* and *Prevotella* species [11] and caries progression can result from acidic and proteolytic activity [10].

The metatranscriptome refers to the gene expression of the whole microbial community [12]. Previous studies have shown differences in microbial gene expression based on sample site within the oral cavity [13] between health and periodontitis [14] and dental caries [15,16]. Microbial gene expression activity in dentin can differ markedly from that in enamel and closer to the tooth surface [16]. These studies focused on dental disease in adults.

The goal of this pilot study was to determine the *in situ* activity of the microbial community and associated species for dental caries in children. Samples representing health and caries at the tooth surface and deep in dentin were examined to represent different disease stages. In caries, one might expect increased gene expression related to acid production [17], whereas in health-associated microbiomes gene expression would reflect that of a stable community able to counter acidic attack [6]. Long-term we sought which metatranscriptome activities characterized different clinical states and so that future studies could use the information in risk assessment and as targets for improved anti-caries therapies.

## Methods

### Study population

Medically healthy 3- to 8-year-old children were recruited from the Community Dental Center in Waterville, Maine. Children in this study had not used antibiotics within the last 3 months, and the parent or guardian consented to the child’s clinical examination and microbial sampling. The children’s primary caregivers completed a survey that incorporated caries risk factors including: demographics, socioeconomic status, dental history and dietary habits [18,19]. The study was performed in full accordance with ethical principles including the World Medical Association Declaration of Helsinki and the study design, protocol, and informed consent were approved by the Institutional Review Board at the Forsyth Institute.

### Clinical characteristics

The caries, plaque and gingival status of children were charted (Table 1). Children were measured at six monthly intervals for up to 2 years and data examined for development of new carious lesions as evidence of caries progression.

**Table 1.** Clinical status of children sampled.

Child	Sample code	Gender	Age (yrs)	dft+ DFT	Caries progess-ing child	Parent has cavities*	Town (Fl+) or well (Fl-) water
**Caries-Free (CF) *n* = 4**							
CF1	8	Girl	3.8	0	0**	1**	Town (Fl+)
CF2	16	Boy	3.8	0	0	0	Town (Fl+)
CF3	11	Girl	4.8	0	0	0	Town (Fl+)
CF4	17	Girl	6.8	0	1	0	Town (Fl+)
**Coronal Caries (CC) *n* = 5**							
CC1	7	Boy	3.4	2	1	1	Well (Fl-)
CC2	18	Boy	4.3	4	1	1	Town (Fl+)
CC3	15	Boy	4.8	6	1	1	Town (Fl+)
CC4	6	Boy	5.2	6	1	1	Well (Fl-)
CC5	2	Girl	6.7	1	1	1	Town (Fl+)
**Dentin Caries (DC) *n* = 5**							
DC1	369 (6)	Boy	6.4	14	1	1	Well (Fl-)
DC2	387	Girl	6.4	9	1	1	Well (Fl-)
DC3	340	Boy	6.8	3	1	1	Well (Fl-)
DC4	348	Boy	7.1	5	1	1	Well (Fl-)
DC5	396	Girl	7.6	5	1	1	Well (Fl-)

* More cavities in parents of caries than caries-free children (*p* < 0.05 Mann-Whitney U test). No significant differences between groups in demographics or diet. ** 0 = No, 1 = Yes, both columns.

### Microbiological methods

Sterile wooden toothpicks were used to collect biofilm samples from supragingival sites of incisors and molars, including from lesions if present, from coronal caries (CC: *n* = 5) and caries-free (CF; *n* = 4) children [20]. Dentin caries (DC: 2 = 5) samples were taken with an excavator during cavity preparation prior to restoring the tooth. Bacterial samples were collected into individual tubes containing 100 µl RNA*later*® (Life technologies, New York). The sample tubes were frozen at −20°C at the clinic and shipped on dry ice to the microbiology laboratory where they were stored at −80°C until processed.

#### Sample preparation

Sample-containing tubes were centrifuged to collect cells and the RNA*later*® was removed by two wash steps with 500 µl DEPC PBS followed by centrifugation at 12,000 × g for 10 min. The following kits and procedures were used to purify RNA following the manufacturer’s instructions: MolYsis® (Molzym GmbH & Co. KG, Bremen, Germany) to remove Eukaryotic DNA, mirVana^TM^ miRNA Isolation Kit (Life Technologies) for RNA extraction, MICROBioEnrich (Life Technologies) for the removal of eukaryotic RNA, MessageAmp^TM^ II-Bacteria RNA amplification kit (Life Technologies) for the amplification of bacterial RNA, and MICROB*Express* (Life Technologies) for the removal of prokaryotic rRNA.

#### Sequencing

Enriched mRNA was prepared for RNA sequencing (RNASeq) using 5 µl of undiluted ‘mRNA’ added to 13 µl of Fragment Prime Finish Mix and the Illumina TruSeq Stranded mRNA Low Sample Protocol was run according to the manufacturer’s instructions (Illumina, San Diego, CA). The prepared samples were run on the Bioanalyzer and their molarity estimated based on the reverse transcription results using the Kapa Library Quantification Kit. The libraries were normalized, pooled and diluted to a 2 ŋM starting concentration. The pooled diluted library was then denatured, diluted, and loaded onto an Illumina NextSeq 500 platform following the manufacturer’s protocol. The samples were sequenced on the NextSeq 500 using Illumina’s High output 150 cycle v2 cartridge.

#### Bioinformatics analysis

The raw RNASeq reads (in FASTQ format) sequenced from the 14 samples were analysed with the software package ‘HUMAnN2’, the HMP Unified Metabolic Analysis Network (http://huttenhower.sph.harvard.edu/humann2) [21]. HUMAnN is a pipeline for efficiently and accurately profiling the presence/absence and abundance of microbial pathways in a community from metagenomic or metatranscriptomic sequencing data. This process, referred to as functional profiling, aims to describe the metabolic potential of a microbial community and its members.

HUMAnN used ‘MetaPhlAn’ [22] to map RNAseq reads to a set of unique clade-specific marker genes identified from ~ 17,000 reference genomes (~ 13,500 bacterial and archaeal, ~ 3,500 viral, and ~ 110 eukaryotic). The reads mapping was performed using ‘Bowtie2’ software [23]. HUMAnN then took the organism-specific gene hits generated by MetaPhlAn and computed the gene family and pathway abundance, and pathway coverage. For comparison, relative abundance of read counts was based on 1 million reads per sample. The results were stratified by organism at the species level, or to genus if not species specific. The gene family definitions were based on the UniRef database [24] and the pathway definitions were based on the MetaCyc database [25].

Stratified species-level transcriptomic profiles were subject to alpha- and beta-diversity analyses, including the rarefaction curves of alpha diversity, principal component analysis (PCoA) beta-diversity of samples among different disease and health groups, and the sequence abundance heatmaps, using the scripts provided by the Quantitative Insights into Microbial Ecology (QIIME) software package [26].

### Statistical analysis

#### Clinical and demographic data

The caries charting measurements were used to calculate total decayed and filled teeth scores for primary (dft) and secondary (DFT) dentitions. A child was considered to have caries progression if a new cavity was detected in the 2-year monitoring period. Clinical and survey (demographic and diet) data were compared between caries-free, caries and dentin groups by the non-parametric statics Kruskal-Wallis test. Mean taxon levels of abundant species and enzymes detected were compared by clinical group using pairwise Mann Whitney U tests (SPSS version 22). Species that are scientifically represented among comparison groups were identified with the Linear Discriminant Analysis Effect Size (LEfSe) software package [22].

## Results

### Study population

Data was analysed from 14 children ranging in age from 3.4 to 7.1 years (Table 1). Six children were girls, and most were within the normal weight range although three of the children sampled were overweight. Children were non-Hispanic, Caucasian, and mothers were the primary caregivers. Samples were obtained from four caries-free children and five children with coronal caries at their first visit, and from six dentin cavities from children at a follow-up visit. One child was sampled at tooth surface and at a later visit from a dentin cavity (#6).

There was no difference in background demographic or dental history of diet data between children in the three groups, except that parents of children with caries all reported having cavities. The children sampled with dentin caries had lower plaque and gingivitis scores, but sampling was at a follow-up (visit 3) when they had received oral hygiene instructions, not the first visit after enrolled in the study. There was no association with overweight status with caries in the sampled children (Table 1).

### Expressed gene sequences mapped to microbial taxa

**Figure 1.**
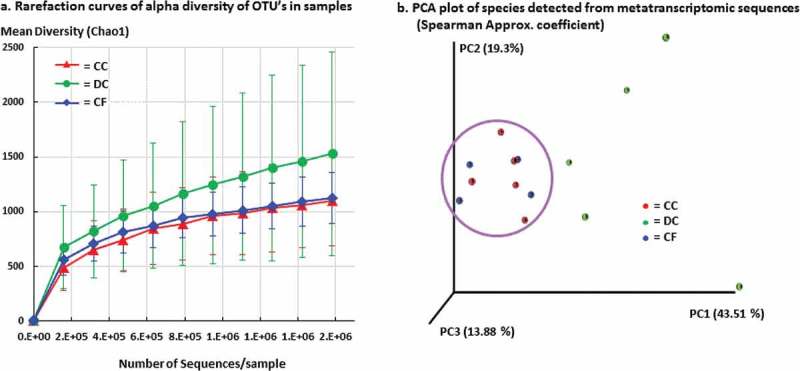
a. Rarefaction curves of alpha diversity in operational taxonomic units (OTU) in samples measured by the Chao1 diversity matrix. b. PCA plot of species detected from rRNA sequences (Spearman Approx. coefficient).

The alpha diversity of gene expression of taxa (operational taxonomic units, OTUs) from samples varied between clinical conditions (Figure 1a). While there was similar diversity in caries-free and coronal caries sites there was increased diversity in the dentin caries. Principal component analysis of species grouped the samples from tooth surfaces, caries-free and coronal caries, together (Figure 1b, purple circle). The dentin samples were quite widely spread from the coronal samples and from each other.

Gene expression sequences mapped to 164 taxa that comprised 108 named species, 12 phylotypes, 33 bacterial genus-level identifications and 13 viruses. The cladogram of LEfSe (Linear discriminant analysis effect size) analysis showed that *Actinobacteria, Neisseriales* and viruses were detected in the dentin caries, *Betaproteobacteria* in caries-free and with one species in *Carnobacteriaceae* that includes lactobacilli in coronal caries (Appendix Figure a). The bar chart of LEfSe analysis illustrated the wide diversity of taxa detected in dentin caries, whereas only taxa in *Carnobacteriaceae* and *Granulicatella* differentiated coronal caries, and *Neisseriaceae, Neisseriales, Betaproteobacteria* and All Bacteria characterized the caries-free samples (Appendix Figure b).

**Figure 2.**
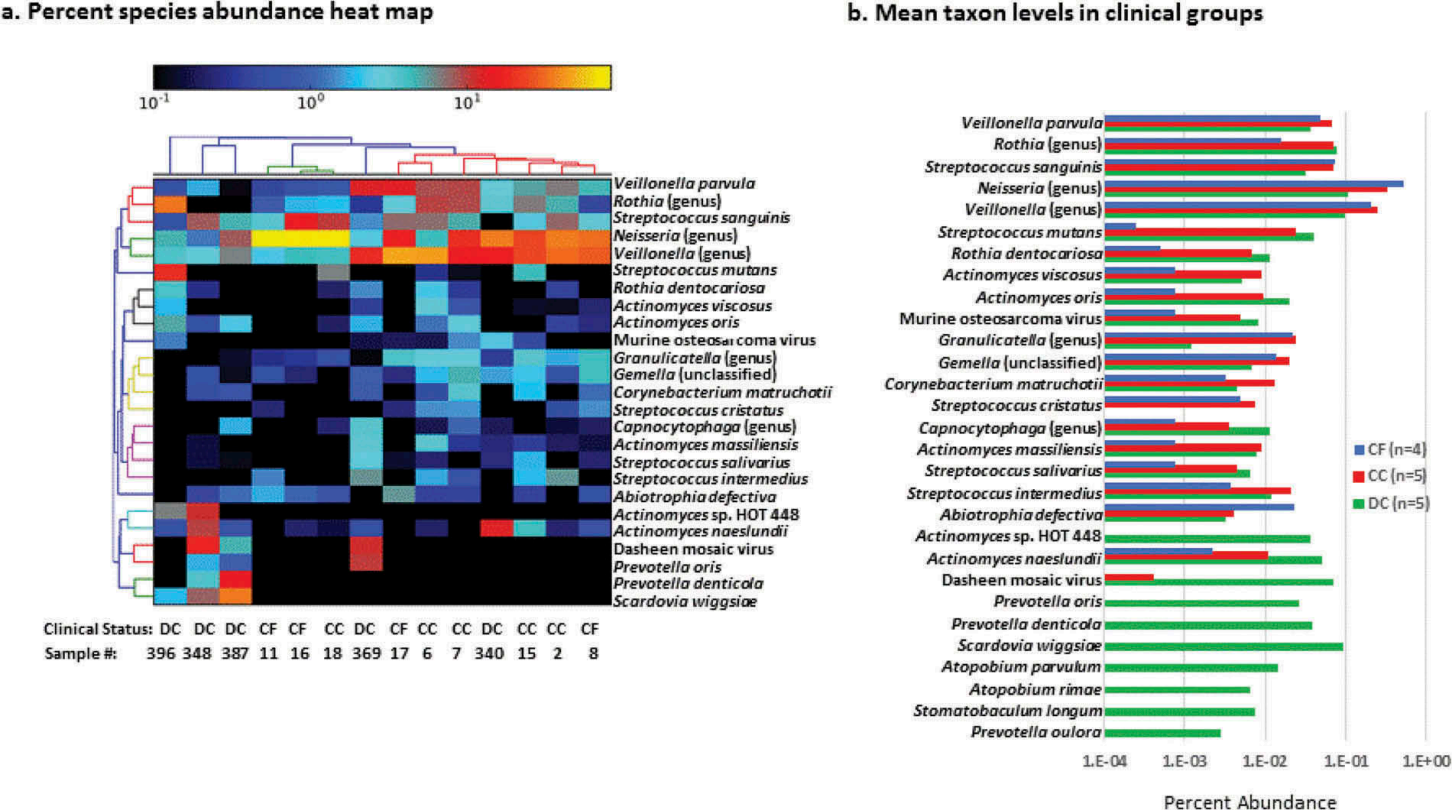
a. Per cent species abundance heat map. b. Mean taxon levels in clinical groups based on 25 abundant species (Figure 2a) with the four next most abundant species detected only in dentin compared by clinical group.

Heat maps of the 25 most abundant taxa (Figure 2a) illustrated a tight cluster (red in top dentrogram) consisting of two caries-free (8,17) with four coronal caries samples (2,6,7,15) dominated by *Neisseria* and *Veillonella* genus level taxa A second group (green in the top dendrogram) of coronal samples (11,16,18), were dominated by *Neisseria* and *Streptococcus sanguinis*. In contrast, bacteria from the dentin caries did not cluster with each other. Species in dentin caries included *Streptococcus mutans* (396), *Scardovia wiggsiae* (348, 389), *Prevotella oris, Actinomyces* and *Veillonella* species (369) or *Actinomyces naeslundii, Veillonella* and *Neisseria* species (340). Additional taxa are in Supplemental Table 1.

Mean per cent abundance of species in each clinical condition (Figure 2b) illustrates similar levels in caries-free and coronal caries samples for many species particularly *Veillonella parvula, S. sanguinis, Streptococcus cristatus* and *Neisseria, Veillonella, Granulicatella* and *Gemella*. Taxa more evident in coronal and dentin caries samples included *S. mutans, Rothia dentocariosa, Actinomyces viscosus, Actinomyces oris, Actinomyces massiliensis, Streptococcus salivarius* and *A. naeslundii* although they were not significantly different. Taxa detected in dentin caries included *Actinomyces* sp. HOT 448, *P. oris, Prevotella denticola, S. wiggsiae, Atopobium parvulum, Atopobium rimae, Stomatobaculum longum* and *Prevotella oulorum.*

### Expressed gene sequences mapped to enzymes (functional profiling)

**Figure 3.**
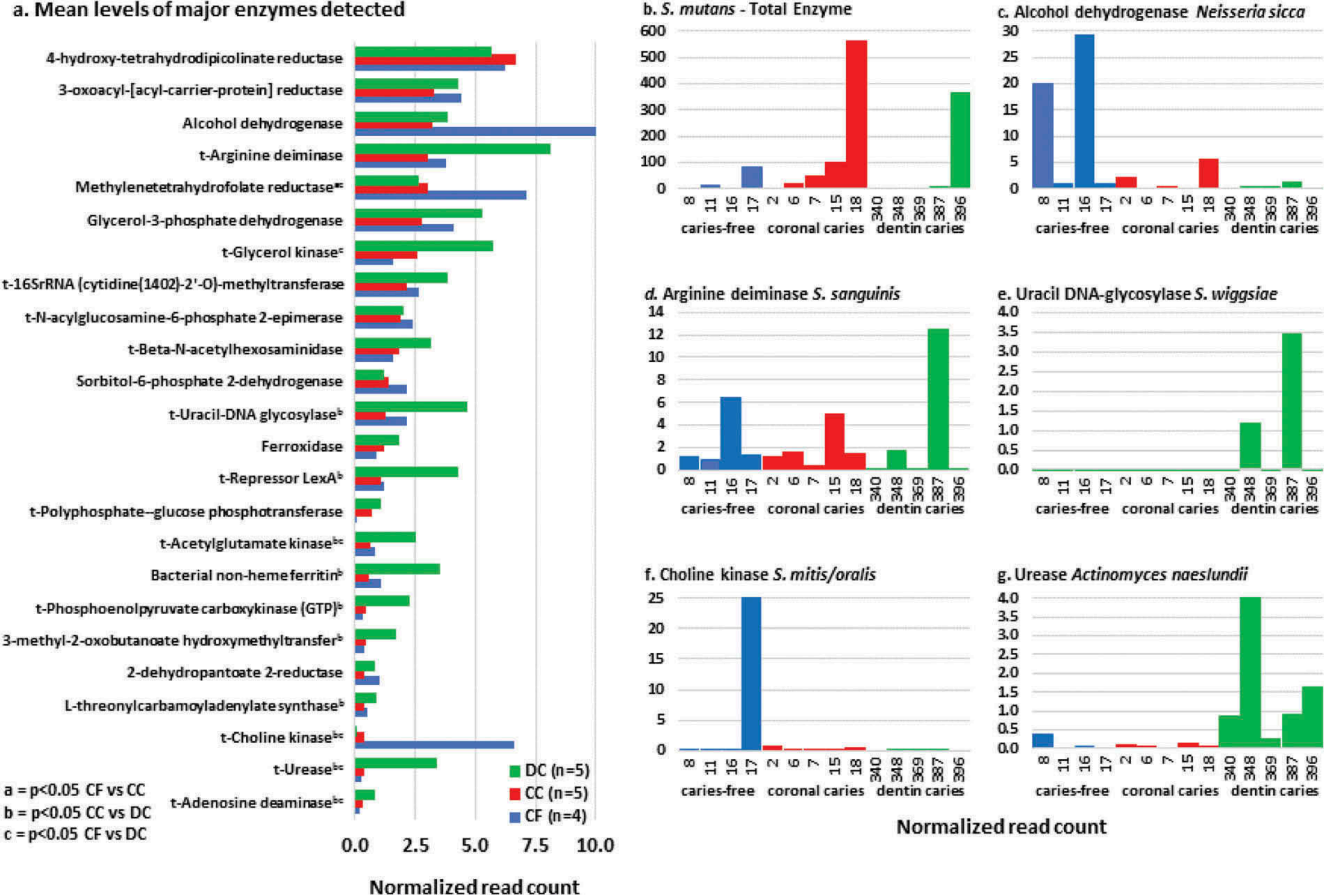
a. Mean levels of enzymes detected in clinical groups (plotted by descending levels in coronal caries based on normalized read counts). b. *S. mutans* total enzyme mapped. c. Alcohol dehydrogenase mapped to *N. sicca*. d. Arginine deiminase mapped to *S. sanguinis*. e. Uracil DNA-glycosylase mapped to *S. wiggsiae*. f. Choline kinase mapped to *S. mitis/oralis/pneumoniae*. g. Urease mapped to *A. naeslundii.*

RNA sequences were mapped in a total of over 1200 genes coding for enzymes. The mean levels of major genes detected in descending order (by read counts normalized by samples) in coronal caries (CC) are given in Figure 3a and supplemental Table 2. At an exploratory threshold (*p* > 0.05 Mann Whitney U test) several genes were elevated in either caries-free or caries (CC and DC). *S. mutans* sequences were mapped mainly to CC (Figure 3b)

***Caries-free (CF)*** Alcohol dehydrogenase mapped mainly to the species *Neisseria sicca* in one (#16) (29.3 units) of the caries-free children (Figure 3c). *S. sanguinis* genes in three of the four caries-free samples accounted to the higher levels of methylenetetrahydrofolate reductase (NAD(P)H). Choline kinase genes were detected in *Streptococcus infantis, Streptococcus mitis (mitis/oralis/pneumoniae)* and *Streptococcus* sp. GMD4S mainly from one caries-free child 17H.

***Coronal caries (CC)*** In coronal caries, the highest levels of genes mapped to species were to *S. mutans* (Figure 3b) and included DNA ligase (ATP) (2.13 × 10^2^ normalized read count), compared with dentin caries (1.10 × 10^2^), and caries-free (8.51 × 10^1^) (data not shown). Next highest genes levels mapped to threonine synthase in *S. mitis (oralis/pneumoniae*) (coronal caries 1.26 × 10^2^; caries-free 7.30 × 10^1^; dentin caries 3.91 × 10^1^).

***Dentin caries (DC)*** Threonine synthase in *S. mitis* was the dominant gene observed for coronal caries (aforementioned). While not significantly different in overall levels between clinical groups, genes for ADS mapped to several species in dentin caries including *S. sanguinis*: dentin caries (DC) 14.3, caries-free (CF) 9.8 and coronal caries (CC) 4.7 (Figure 3d), *A. naeslundii* (DC 3.0, CC 0.6, CF 0.5) and *A. massiliensis* (DC 3.0, CC 0.3, CF 0.04). Other taxa with ADS genes in only dentin caries included *S. wiggsiae* (DC 4.0), *Streptococcus australis* (DC 1.9) and *A. rimae* (DC 1.4). Genes to glycerol kinase mapped mainly to *S. sanguinis* but levels were similar between clinical groups (DC 3.5, CC 3.9, CF 2.7). Other taxa that glycerol kinase genes mapped to in dentin caries included: *Rothia aeria* (DC 3.4, CC 1.6, CF 0.4), *P. denticola* (DC 2.4) *R. dentocariosa* (DC 2.1, CC 1.5, CF 0.1) and *S. australis* (DC 1.1, CC 0.1).

Uracil-DNA glycosylase genes mapped mainly to *P. denticola* (DC 5.0), *A. naeslundii* (DC 1.3, CC 0.1, CF 0.1) (Figure 3e) and *Actinomyces* sp. HOT 448 (DC 1.1). Repressor LexA genes detected mapped principally to *S. wiggsiae* (DC 5.0), *Dialister invisus* (DC 2.1) and *V. parvula* (DC 1.6; CC 1.5, CF 1.4). Acetylglutamate kinase genes in dentin caries mapped mainly to *A. naeslundii* (DC 1.9; CC 0.4, CF 0.1), *V. parvula* (DC 1.6; CC 0.3, CF 0.5) and *A. massiliensis* (DC 1.3, CC 0.3, CF 0.1). Bacterial non-heme ferritin genes mapped to *Haemophilus parainfluenzae* (DC 5.8, CC 0.1, CF 0.4), *P. denticola* (DC 2.1), *D. invisus* (DC 1.8) and *R. dentocariosa* (DC 1.0, CC 1.1, CF 0.1). Phosphoenolpyruvate carboxykinase (GTP) genes mapped to *Actinomyces* sp. HOT 448 (DC 3.6) and *A. naeslundii* (DC 1.8, CC 0.6, CF 0.4). 3-methyl-2-oxobutanoate hydroxymethyltransferase genes mapped to *Lachnoanaerobaculum saburreum* (DC 0.2). L-threonylcarbamoyladenylate synthase genes mapped principally to *V. parvula* (DC 1.7, CC 1.1, CF 0.8). Choline kinase genes mapped mainly to one CF sample (Figure 3e). Urease genes mapped mainly to: *A. naeslundii* (DC 7.8; CC 0.3, CF 0.4) (Figure 3f) and *A. viscosus* (DC 5.1, CC 0.6, CF 0.4). Adenosine deaminase genes mapped principally to *Actinomyces* sp. HOT 448 (DC 0.6), *A. parvulum* (DC 0.5) and *A. massiliensis* (DC 0.4, CC 0.1, CF 0.1).

## Discussion

The ability to examine functional profiling of the oral biofilm community focuses on addressing the question ‘What are the microbes in my community-of-interest doing (or capable of doing)?’ in addition to providing a new approach to examine the oral microbiota in health and disease [27]. Compared with 16S profiling, metatranscriptomic analysis can provide a better picture of the species that are metabolically active in different caries types[11] and thus revealing the community function which is more relevant to disease states. This approach revealed species identifications consistent with bacterial identifications from samples using culture and molecular methods, providing a measure of validity to the functional profiling approach to describing the microbiota. Considering genes from *S. mutans* and *S. wiggsiae*, while *S. mutans* was elevated in coronal and dentin caries compared with caries-free, *S. wiggsiae* was associated with dentin caries suggesting that *S. wiggsiae* was primarily active in dentin lesions. This latest information adds to our knowledge of the ecology of *S. wiggsiae* in dental caries. While there were few differences in gene expression between the coronal caries and caries-free samples, there was increased and more diversity in genes detected in dentin caries.

### Microbiota differences

#### Sample site

The genes detected in coronal caries and caries-free children clustered together suggesting an overlap in gene expression of the microbiotas on the tooth surface. This contrasted with the genes of dentin caries, which differed from each other and reflected a wider range of metabolic capabilities. Differences in the microbiome composition from enamel (coronal) to dentin caries and increased diversity in dentin caries was observed in a metagenomic analysis [13] as in the current report. The pH of dentin caries was found to vary considerably ranging from pH 4.5 to over pH 7, with the different microbiotas being associated with various levels of acidity [28], consistent with the diversity in the microbiota in dentin caries sites observed of the current report.

#### Health and disease

Detection of genes in *S. mutans, R. dentocariosa*, several *Actinomyces* species and *S. salivarius* in coronal caries compared with caries-free sites is consistent with other reports [5,29–32]. Detection of genes to these species fit with the expanded ecological plaque hypotheses that proposes blooming of *Actinomyces* and non-mutans streptococci in early stages of dental caries [17]. Under this hypothesis, these species set up the environment for more acid-tolerant and acidogenic species that would include *S. mutans, S. wiggsiae* [33], *Actinomyces* sp. HOT 448 [34] observed in dentin caries in the current report. *S. wiggsiae* is a relatively newly recognized species that has been detected in caries in several reports from the US [5], Europe [35] and Brazil [36]. Other highly acidogenic, lactate-producing species in dentin included *A. rimae* and *A. parvulum* [37] although the acid tolerance of these species is unknown. Other less acidogenic species with genes detected in dentin, included *Prevotella* and *Capnocytophaga* species and *S. longum* [38], likely reflecting the higher pH deeper in dentin compared to the tooth surface, and proteolytic activity related to dentin caries [10]. Detection of genes in dentin mapping to *A. oris, P. denticola, A. parvulum* and the genus *Rothia* in dentin was as previously reported [11]. *Abiotrophia defectiva* was detected in caries-free sites, although not significantly associated with health. This previously ‘aberrant *Streptococcus* species’ is clinically associated with bacterial endocarditis and has been observed in health compared with early childhood caries [39] as in the current study.

*Viruses*. Genes mapped to several viral taxa as previously described [27]. In the current report the principal viruses detected from gene expression sequences were the murine osteosarcoma virus and the dasheen mosaic virus which is a plant virus. It is not clear what their role, if any, may be in dental caries, or oral pathology.

### Functional profiles – community gene expression

The greater diversity of genes that was detected in dentin compared with caries-free or caries samples from coronal sites was linked with detection of more enzymes and taxa in dentin. This is consistent with observations of acidogenic and proteolytic activity in dentin caries [10,40].

#### Health and disease

Alcohol dehydrogenase, methylenetetrahydrofolate reductase, and choline kinase genes were detected at higher levels in caries-free compared to coronal or dentin caries. Alcohol dehydrogenase mapped mainly to *N. sicca* which produces this enzyme [41], Alcohol dehydrogenase can play a role in neutralizing bacterial acid production through reduction of nicotinamide adenine dinucleotide (NAD^+^ to NADH). This suggests an alternative mechanism to NH_3_ production to counteract community bacterial acid in health. Genes for methylenetetrahydrofolate reductase, which is involved in methionine production, mapped principally to *S. sanguinis* and while the gene has been detected in bacteria no specific role in the oral biofilm community was found. Choline kinase genes mapped mainly to *S. mitis* as previously reported [42] and are considered to play a role in cell wall lysis. It is not clear what their role in caries-free sites would be.

The highest gene expression was in dentin caries including for ADS, glycerol kinase, uracil-DNA glycosylase and urease. ADS and urease have been associated with reduced caries activity based on ammonia production and raising the pH to counterbalance acidic conditions typical of active caries [43]. It seems likely these enzymes also involved in the higher pH found deep in in dentin compared to the tooth surface [28]. Linking ADS with *S. sanguinis* and urease with *A. naeslundii* in the microbial community is consistent with the metabolic properties of these species.

Glycerol kinase is part of the glycolysis pathway rather than that for refined carbohydrates as reported for dentin caries [44]. The roles of the other enzymes in caries is unclear.

The functional profiling approach used in this study could identify potential gene activity and associated species and indicate likely community activity in health and disease both previously recognized or reveal new enzyme activities. ‘ Next generation RNA sequencing (RNA-seq)’ provides an unbiased and culture-free method to study the gene expression of a microbial community. The high depth of Illumina sequencing makes it robust to identify genes expressed a low level but play key roles in regulating metabolic functions and pathways. The relatively high cost associated with the Illumina sequencing has been a limitation for this technology, but costs have been reducing with the popularity and the increasing high throughput of this approach. This renders Illumina sequencing possible for metatranscriptomic studies as in this report.

A major limitation of the current report was the small number of sites examined so it is unclear how the findings are applicable overall. Further, unlike study of the transcriptome from bacterial culture that allows for a sufficient amount of RNA, the small clinical samples size led to limited amounts of RNA that might also impact the results. The transcriptome profile might only reveal the relatively abundant functionality, but not that of the less abundant, but nevertheless could potentially have more biologically significant impact. Moreover, the resultant DNA yield from the small sample sizes precluded simultaneous metagenome analysis. Additionally, samples were collected without consideration of food intake, and if enzyme activity related acid production was limited or enhanced to periods just after fermentable carbohydrate intake that could have been missed. We suggest future study to consider timing of cariogenic food intakes particularly for coronal caries.

## Conclusion

Metatranscriptomic analysis by functional profiling confirmed previous observations about key bacteria and cariogenic mechanisms that were previously known, and revealed some new enzyme activities by species in both caries and caries-free communities. Comparing coronal and dentin caries clarified the marked differences between these communities in both bacterial composition and functional profiles.

## Appendix

**Table 1.** Mean levels of species/taxa in caries-free (CF), coronal caries (CC) and dentin caries (DC).

Family	Species/Taxon	CF (*n* = 4)	CF SEM	CC (*n* = 5)	CC SEM	DC (*n* = 6)	DC SEM
Actinobacteria	*Actinobaculum* sp. HOT 183	**0.00E+ 00**	0.00E+ 00	**0.00E+ 00**	0.00E+ 00	**2.10E-02**	2.10E-02
	*Actinobaculum* genus	**2.50E-04**	2.50E-04	**8.00E-04**	3.74E-04	**4.00E-03**	3.27E-03
	*Actinomyces georgiae*	**0.00E+ 00**	0.00E+ 00	**2.00E-04**	2.00E-04	**8.00E-04**	8.00E-04
	*Actinomyces johnsonii*	**5.00E-04**	5.00E-04	**0.00E+ 00**	0.00E+ 00	**6.60E-03**	4.28E-03
	*Actinomyces massiliensis*	**7.50E-04**	4.79E-04	**8.80E-03**	6.58E-03	**7.60E-03**	6.62E-03
	*Actinomyces naeslundii*	**2.25E-03**	1.65E-03	**1.06E-02**	8.85E-03	**4.88E-02**	2.66E-02
	*Actinomyces odontolyticus*	**1.00E-03**	1.00E-03	**2.00E-04**	2.00E-04	**0.00E+ 00**	0.00E+ 00
	*Actinomyces oris*	**7.50E-04**	7.50E-04	**9.20E-03**	5.12E-03	**1.96E-02**	8.86E-03
	*Actinomyces* sp. HOT 448	**0.00E+ 00**	0.00E+ 00	**0.00E+ 00**	0.00E+ 00	**3.60E-02**	2.33E-02
	*Actinomyces viscosus*	**7.50E-04**	4.79E-04	**8.80E-03**	6.20E-03	**5.00E-03**	3.56E-03
	*Brevibacterium* genus	**0.00E+ 00**	0.00E+ 00	**2.00E-04**	2.00E-04	**0.00E+ 00**	0.00E+ 00
	*Corynebacterium durum*	**1.00E-03**	1.00E-03	**1.00E-03**	6.32E-04	**4.40E-03**	2.32E-03
	*Corynebacterium matruchotii*	**3.25E-03**	2.93E-03	**1.28E-02**	7.28E-03	**4.40E-03**	1.91E-03
	*Rothia aeria*	**3.00E-03**	2.04E-03	**4.00E-03**	1.10E-03	**1.46E-02**	1.03E-02
	*Rothia dentocariosa^a^*	**5.00E-04**	2.89E-04	**6.80E-03**	4.09E-03	**1.10E-02**	7.22E-03
	*Rothia mucilaginosa*	**5.00E-04**	5.00E-04	**4.00E-04**	2.45E-04	**3.60E-03**	2.20E-03
	*Rothia* genus^a^	**1.55E-02**	5.75E-03	**6.78E-02**	1.51E-02	**7.54E-02**	6.64E-02
	*Propionibacterium acnes*	**2.50E-04**	2.50E-04	**6.00E-04**	4.00E-04	**0.00E+ 00**	0.00E+ 00
	*Propionibacterium propionicum*	**0.00E+ 00**	0.00E+ 00	**0.00E+ 00**	0.00E+ 00	**1.00E-03**	6.32E-04
	*Bifidobacterium dentium*	**0.00E+ 00**	0.00E+ 00	**0.00E+ 00**	0.00E+ 00	**1.60E-03**	1.60E-03
	*Scardovia* genus	**0.00E+ 00**	0.00E+ 00	**0.00E+ 00**	0.00E+ 00	**1.42E-02**	1.42E-02
	*Scardovia wiggsiae^d^*	**0.00E+ 00**	0.00E+ 00	**0.00E+ 00**	0.00E+ 00	**9.04E-02**	6.71E-02
	*Atopobium parvulum^d^*	**0.00E+ 00**	0.00E+ 00	**0.00E+ 00**	0.00E+ 00	**6.40E-03**	3.08E-03
	*Atopobium rimae^d^*	**0.00E+ 00**	0.00E+ 00	**0.00E+ 00**	0.00E+ 00	**1.70E-02**	1.35E-02
	*Cryptobacterium curtum*	**0.00E+ 00**	0.00E+ 00	**0.00E+ 00**	0.00E+ 00	**2.40E-03**	1.47E-03
	*Olsenella* genus	**0.00E+ 00**	0.00E+ 00	**0.00E+ 00**	0.00E+ 00	**2.00E-04**	2.00E-04
Bacteroidetes	*Porphyromonas catoniae*	**1.23E-02**	1.23E-02	**0.00E+ 00**	0.00E+ 00	**0.00E+ 00**	0.00E+ 00
	*Alloprevotella tannerae*	**0.00E+ 00**	0.00E+ 00	**0.00E+ 00**	0.00E+ 00	**4.00E-04**	4.00E-04
	*Alloprevotella genus*	**0.00E+ 00**	0.00E+ 00	**0.00E+ 00**	0.00E+ 00	**6.00E-04**	6.00E-04
	*Prevotella denticola*	**0.00E+ 00**	0.00E+ 00	**0.00E+ 00**	0.00E+ 00	**3.68E-02**	2.85E-02
	*Prevotella melaninogenica*	**0.00E+ 00**	0.00E+ 00	**0.00E+ 00**	0.00E+ 00	**6.00E-04**	4.00E-04
	*Prevotella nigrescens^d^*	**0.00E+ 00**	0.00E+ 00	**0.00E+ 00**	0.00E+ 00	**2.80E-03**	1.53E-03
	*Prevotella oris^d^*	**0.00E+ 00**	0.00E+ 00	**0.00E+ 00**	0.00E+ 00	**2.64E-02**	1.99E-02
	*Prevotella oulorum^d^*	**0.00E+ 00**	0.00E+ 00	**0.00E+ 00**	0.00E+ 00	**4.20E-03**	2.06E-03
	*Prevotella pallens*	**0.00E+ 00**	0.00E+ 00	**0.00E+ 00**	0.00E+ 00	**2.00E-04**	2.00E-04
	*Prevotella salivae*	**0.00E+ 00**	0.00E+ 00	**0.00E+ 00**	0.00E+ 00	**2.00E-04**	2.00E-04
	*Prevotella veroralis*	**0.00E+ 00**	0.00E+ 00	**0.00E+ 00**	0.00E+ 00	**3.80E-03**	3.80E-03
	*Capnocytophaga gingivalis*	**2.50E-04**	2.50E-04	**0.00E+ 00**	0.00E+ 00	**6.00E-04**	4.00E-04
	*Capnocytophaga* genus	**7.50E-04**	4.79E-04	**3.40E-03**	1.94E-03	**1.12E-02**	7.06E-03
	*Riemerella* genus	**0.00E+ 00**	0.00E+ 00	**0.00E+ 00**	0.00E+ 00	**2.00E-04**	2.00E-04
TM7	TM7 TM7b	**0.00E+ 00**	0.00E+ 00	**4.00E-03**	3.10E-03	**4.00E-04**	4.00E-04
	TM7 TM7c	**0.00E+ 00**	0.00E+ 00	**3.80E-03**	2.46E-03	**2.00E-04**	2.00E-04
Firmicutes	*Gemella haemolysans*	**1.75E-03**	1.75E-03	**3.20E-03**	1.66E-03	**2.00E-04**	2.00E-04
	*Gemella morbillorum*	**2.50E-04**	2.50E-04	**2.00E-04**	2.00E-04	**0.00E+ 00**	0.00E+ 00
	*Gemella sanguinis*	**0.00E+ 00**	0.00E+ 00	**0.00E+ 00**	0.00E+ 00	**6.00E-04**	4.00E-04
	*Gemella* genus	**1.38E-02**	1.01E-02	**1.92E-02**	8.58E-03	**6.80E-03**	3.07E-03
	*Abiotrophia defectiva^ad^*	**2.25E-02**	1.14E-02	**4.00E-03**	9.49E-04	**3.20E-03**	2.08E-03
	*Granulicatella adiacens*	**3.25E-03**	2.93E-03	**4.00E-03**	1.92E-03	**2.00E-04**	2.00E-04
	*Granulicatella elegans*	**5.00E-04**	5.00E-04	**0.00E+ 00**	0.00E+ 00	**0.00E+ 00**	0.00E+ 00
	*Granulicatella* genus^cd^	**2.20E-02**	1.10E-02	**2.36E-02**	5.73E-03	**1.20E-03**	7.35E-04
	*Streptococcus australis*	**0.00E+ 00**	0.00E+ 00	**2.00E-04**	2.00E-04	**3.00E-03**	1.90E-03
	*Streptococcus cristatus*	**5.00E-03**	3.70E-03	**7.20E-03**	3.51E-03	**0.00E+ 00**	0.00E+ 00
	*Streptococcus gordonii*	**0.00E+ 00**	0.00E+ 00	**4.00E-04**	4.00E-04	**6.00E-04**	6.00E-04
	*Streptococcus infantis*	**7.50E-04**	7.50E-04	**4.00E-04**	4.00E-04	**8.00E-04**	5.83E-04
	*Streptococcus intermedius*	**3.75E-03**	3.42E-03	**2.04E-02**	9.87E-03	**1.16E-02**	1.16E-02
	*Streptococcus mitis/ oralis/pneumoniae*	**2.00E-03**	5.77E-04	**8.80E-03**	5.23E-03	**8.00E-04**	3.74E-04
	*Streptococcus mutans*	**2.50E-04**	2.50E-04	**2.32E-02**	1.38E-02	**3.86E-02**	3.86E-02
	*Streptococcus parasanguinis*	**1.75E-03**	1.03E-03	**2.00E-04**	2.00E-04	**2.40E-03**	1.91E-03
	*Streptococcus salivarius*	**7.50E-04**	4.79E-04	**4.40E-03**	3.92E-03	**6.40E-03**	4.93E-03
	*Streptococcus sanguinis*	**7.40E-02**	2.77E-02	**6.74E-02**	1.35E-02	**3.10E-02**	1.59E-02
	*Streptococcus* sp. AS14	**0.00E+ 00**	0.00E+ 00	**0.00E+ 00**	0.00E+ 00	**3.20E-03**	3.20E-03
	*Streptococcus* sp. BS35b	**0.00E+ 00**	0.00E+ 00	**2.80E-03**	2.80E-03	**0.00E+ 00**	0.00E+ 00
	*Streptococcus* sp. GMD4S	**0.00E+ 00**	0.00E+ 00	**0.00E+ 00**	0.00E+ 00	**1.20E-03**	1.20E-03
	*Eubacterium infirmum*	**0.00E+ 00**	0.00E+ 00	**0.00E+ 00**	0.00E+ 00	**2.00E-03**	2.00E-03
	*Eubacterium brachy*	**2.50E-04**	2.50E-04	**0.00E+ 00**	0.00E+ 00	**0.00E+ 00**	0.00E+ 00
	*Ruminococcus torques*	**0.00E+ 00**	0.00E+ 00	**0.00E+ 00**	0.00E+ 00	**0.00E+ 00**	0.00E+ 00
	*Johnsonella ignava*	**5.00E-04**	2.89E-04	**0.00E+ 00**	0.00E+ 00	**0.00E+ 00**	0.00E+ 00
	*Lachnoanaerobaculum saburreum*	**2.50E-04**	2.50E-04	**0.00E+ 00**	0.00E+ 00	**4.00E-04**	4.00E-04
	*Lachnospiraceae* sp. HOT 107	**2.50E-04**	2.50E-04	**0.00E+ 00**	0.00E+ 00	**0.00E+ 00**	0.00E+ 00
	*Oribacterium* sp. HOT 078^bcd^	**0.00E+ 00**	0.00E+ 00	**0.00E+ 00**	0.00E+ 00	**7.40E-03**	4.20E-03
	*Stomatobaculum longum*	**0.00E+ 00**	0.00E+ 00	**0.00E+ 00**	0.00E+ 00	**1.40E-03**	6.78E-04
	*Peptostreptococcus* genus	**0.00E+ 00**	0.00E+ 00	**0.00E+ 00**	0.00E+ 00	**4.00E-04**	4.00E-04
	*Dialister invisus*	**0.00E+ 00**	0.00E+ 00	**0.00E+ 00**	0.00E+ 00	**1.48E-02**	1.48E-02
	*Megasphaera micronuciformis*	**0.00E+ 00**	0.00E+ 00	**0.00E+ 00**	0.00E+ 00	**3.00E-03**	2.14E-03
	*Mitsuokella* genus	**0.00E+ 00**	0.00E+ 00	**2.00E-04**	2.00E-04	**2.00E-04**	2.00E-04
	*Selenomonas artemidis*	**0.00E+ 00**	0.00E+ 00	**0.00E+ 00**	0.00E+ 00	**8.00E-04**	8.00E-04
	*Selenomonas noxia*	**2.25E-03**	2.25E-03	**1.40E-03**	9.80E-04	**2.00E-03**	8.37E-04
	*Veillonella atypica*	**0.00E+ 00**	0.00E+ 00	**0.00E+ 00**	0.00E+ 00	**4.00E-04**	4.00E-04
	*Veillonella parvula*	**4.78E-02**	2.98E-02	**6.50E-02**	1.63E-02	**3.62E-02**	2.30E-02
	*Veillonella* genus	**2.06E-01**	1.06E-01	**2.44E-01**	6.68E-02	**9.50E-02**	3.24E-02
Fusobacteria	*Fusobacterium nucleatum*	**0.00E+ 00**	0.00E+ 00	**1.00E-03**	7.75E-04	**5.20E-03**	4.47E-03
	*Fusobacterium periodonticum*	**0.00E+ 00**	0.00E+ 00	**0.00E+ 00**	0.00E+ 00	**6.00E-04**	4.00E-04
	*Leptotrichia goodfellowii*	**0.00E+ 00**	0.00E+ 00	**0.00E+ 00**	0.00E+ 00	**6.00E-04**	6.00E-04
	*Leptotrichia* genus	**5.00E-04**	2.89E-04	**2.80E-03**	2.56E-03	**1.00E-03**	1.00E-03
Proteobacteria	*Rhodopseudomonas palustris*	**5.00E-04**	5.00E-04	**1.80E-03**	1.20E-03	**2.00E-04**	2.00E-04
	*Rhodopseudomonas* genus	**2.50E-04**	2.50E-04	**0.00E+ 00**	0.00E+ 00	**0.00E+ 00**	0.00E+ 00
	*Lautropia mirabilis^d^*	**0.00E+ 00**	0.00E+ 00	**0.00E+ 00**	0.00E+ 00	**3.00E-03**	2.05E-03
	*Thiomonas* genus	**0.00E+ 00**	0.00E+ 00	**4.00E-04**	4.00E-04	**0.00E+ 00**	0.00E+ 00
	*Kingella denitrificans*	**2.50E-04**	2.50E-04	**2.00E-04**	2.00E-04	**0.00E+ 00**	0.00E+ 00
	*Kingella oralis*	**1.25E-03**	9.46E-04	**1.40E-03**	7.48E-04	**0.00E+ 00**	0.00E+ 00
	*Kingella* genus	**1.38E-02**	1.15E-02	**5.60E-03**	1.57E-03	**2.40E-03**	2.16E-03
	*Neisseria elongata*	**1.25E-03**	9.46E-04	**3.00E-03**	1.48E-03	**1.60E-03**	1.60E-03
	*Neisseria flavescens*	**0.00E+ 00**	0.00E+ 00	**0.00E+ 00**	0.00E+ 00	**4.00E-04**	2.45E-04
	*Neisseria macacae*	**0.00E+ 00**	0.00E+ 00	**4.00E-04**	4.00E-04	**0.00E+ 00**	0.00E+ 00
	*Neisseria meningitidis*	**2.50E-04**	2.50E-04	**4.00E-04**	4.00E-04	**8.00E-04**	5.83E-04
	*Neisseria sicca*	**4.00E-03**	2.04E-03	**1.40E-03**	7.48E-04	**4.00E-04**	2.45E-04
	*Neisseria subflava*	**0.00E+ 00**	0.00E+ 00	**0.00E+ 00**	0.00E+ 00	**2.00E-04**	2.00E-04
	*Neisseria* genus	**5.17E-01**	1.61E-01	**3.15E-01**	1.10E-01	**1.05E-01**	6.38E-02
	*Campylobacter gracilis*	**0.00E+ 00**	0.00E+ 00	**2.00E-04**	2.00E-04	**3.80E-03**	3.56E-03
	*Campylobacter showae*	**0.00E+ 00**	0.00E+ 00	**0.00E+ 00**	0.00E+ 00	**4.00E-04**	4.00E-04
	*Cardiobacterium hominis*	**1.50E-03**	6.45E-04	**8.00E-04**	3.74E-04	**0.00E+ 00**	0.00E+ 00
	*Cardiobacterium valvarum*	**2.50E-04**	2.50E-04	**0.00E+ 00**	0.00E+ 00	**0.00E+ 00**	0.00E+ 00
	*Enterobacter cloacae*	**0.00E+ 00**	0.00E+ 00	**0.00E+ 00**	0.00E+ 00	**2.00E-04**	2.00E-04
	*Escherichia coli*	**5.00E-04**	2.89E-04	**1.00E-03**	1.00E-03	**4.00E-04**	2.45E-04
	*Escherichia* genus	**0.00E+ 00**	0.00E+ 00	**4.00E-04**	4.00E-04	**2.00E-04**	2.00E-04
	*Pantoea* genus	**2.50E-04**	2.50E-04	**0.00E+ 00**	0.00E+ 00	**0.00E+ 00**	0.00E+ 00
	*Actinobacillus* genus	**0.00E+ 00**	0.00E+ 00	**0.00E+ 00**	0.00E+ 00	**2.00E-04**	2.00E-04
	*Aggregatibacter* genus	**0.00E+ 00**	0.00E+ 00	**6.00E-04**	6.00E-04	**0.00E+ 00**	0.00E+ 00
	*Haemophilus parainfluenzae*	**7.50E-04**	4.79E-04	**2.00E-04**	2.00E-04	**1.10E-02**	3.00E-03
	*Pasteurella* genus	**0.00E+ 00**	0.00E+ 00	**0.00E+ 00**	0.00E+ 00	**2.00E-04**	2.00E-04
	*Acinetobacter* genus	**2.00E-03**	2.00E-03	**6.00E-04**	4.00E-04	**0.00E+ 00**	0.00E+ 00
	*Pseudomonas* genus	**0.00E+ 00**	0.00E+ 00	**8.00E-04**	8.00E-04	**1.20E-03**	9.70E-04
	*Vibrio cholerae^cd^*	**0.00E+ 00**	0.00E+ 00	**0.00E+ 00**	0.00E+ 00	**7.80E-03**	3.72E-03
Treponema	*Treponema socranskii*	**0.00E+ 00**	0.00E+ 00	**0.00E+ 00**	0.00E+ 00	**6.00E-04**	4.00E-04
Naumovozyma	*Naumovozyma* genus	**0.00E+ 00**	0.00E+ 00	**2.00E-04**	2.00E-04	**0.00E+ 00**	0.00E+ 00
Yeast	*Candida* genus	**0.00E+ 00**	0.00E+ 00	**2.00E-04**	2.00E-04	**0.00E+ 00**	0.00E+ 00
Viruses	Hpunalikevirus Vibrio phage kappa (DNA)	**0.00E+ 00**	0.00E+ 00	**0.00E+ 00**	0.00E+ 00	**1.26E-02**	1.26E-02
	Mastadenovirus Human adenovirus E (DNA)	**0.00E+ 00**	0.00E+ 00	**1.04E-02**	1.02E-02	**0.00E+ 00**	0.00E+ 00
	Bromovirus Brome mosaic virus (RNA)	**0.00E+ 00**	0.00E+ 00	**0.00E+ 00**	0.00E+ 00	**6.00E-04**	6.00E-04
	Endornavirus Bell pepper endornavirus (RNA)	**0.00E+ 00**	0.00E+ 00	**2.00E-04**	2.00E-04	**0.00E+ 00**	0.00E+ 00
	Narnavirus Saccharomyces 20S RVA narnavirus (RNA)	**0.00E+ 00**	0.00E+ 00	**8.00E-04**	8.00E-04	**0.00E+ 00**	0.00E+ 00
	Potyvirus Dasheen mosaic virus^d^ (RNA)	**0.00E+ 00**	0.00E+ 00	**4.00E-04**	2.45E-04	**6.82E-02**	3.45E-02
	Alpharetrovirus Avian myelocytomatosis virus (RNA)	**0.00E+ 00**	0.00E+ 00	**0.00E+ 00**	0.00E+ 00	**2.00E-04**	2.00E-04
	Gammaretrovirus Murine osteosarcoma virus (RNA)	**7.50E-04**	4.79E-04	**4.80E-03**	2.65E-03	**8.00E-03**	4.64E-03
	Tobamovirus Cucumber green mottle mosiac virus (RNA)	**0.00E+ 00**	0.00E+ 00	**0.00E+ 00**	0.00E+ 00	**1.80E-03**	1.80E-03

*p* ≤ 0.05, a = CF vs CC, b = CF vs DC, c = CC vs DC, d = all groups.

**Table 2.** Mean levels of enzymes detected in caries-free (CF), coronal caries (CC) and dentin caries (DC).

Enzymes	CF (*n* = 4)	CF SEM	CC (*n* = 5)	CC SEM	DC (*n* = 6)	DC SEM
Alcohol dehydrogenase	**1.01E+ 01**	8.35E+ 00	**3.21E+ 00**	1.45E+ 00	**3.79E+ 00**	9.54E-01
3-oxoacyl-[acyl-carrier-protein] reductase	**4.43E+ 00**	5.82E-01	**3.27E+ 00**	1.19E+ 00	**4.24E+ 00**	1.74E+ 00
L-threonine 3-dehydrogenase	**2.55E-02**	1.59E-02	**1.84E-02**	1.15E-02	**3.96E-02**	1.47E-02
All-trans-retinol dehydrogenase (NAD(+))	**0.00E+ 00**	0.00E+ 00	**0.00E+ 00**	0.00E+ 00	**1.20E-01**	1.09E-01
2-deoxy-D-gluconate 3-dehydrogenase	**0.00E+ 00**	0.00E+ 00	**0.00E+ 00**	0.00E+ 00	**2.82E-02**	2.82E-02
3-dehydro-L-gulonate 2-dehydrogenase	**6.00E-03**	6.00E-03	**1.40E-03**	1.40E-03	**3.72E-02**	3.01E-02
GDP-mannose 6-dehydrogenase	**0.00E+ 00**	0.00E+ 00	**0.00E+ 00**	0.00E+ 00	**8.68E-02**	8.68E-02
dTDP-4-dehydrorhamnose reductase	**4.57E-01**	1.89E-01	**2.76E-01**	8.88E-02	**1.49E+ 00**	6.77E-01
Mannitol 2-dehydrogenase (NADP(+))	**9.45E-02**	9.45E-02	**0.00E+ 00**	0.00E+ 00	**3.38E-02**	3.38E-02
Sorbitol-6-phosphate 2-dehydrogenase	**2.18E+ 00**	6.35E-01	**1.34E+ 00**	6.21E-01	**1.16E+ 00**	8.32E-01
Ureidoglycolate dehydrogenase	**6.75E-03**	6.75E-03	**4.00E-03**	4.00E-03	**2.80E-02**	2.65E-02
3-hydroxybutyryl-CoA dehydrogenase	**6.23E-02**	5.77E-02	**1.19E-01**	6.96E-02	**4.72E-01**	2.47E-01
2-dehydropantoate 2-reductase	**1.04E+ 00**	2.36E-01	**3.74E-01**	9.35E-02	**7.78E-01**	3.16E-01
Mannitol-1-phosphate 5-dehydrogenase	**9.98E-02**	5.39E-02	**1.14E-01**	6.64E-02	**4.39E-01**	1.24E-01
D-xylose 1-dehydrogenase (NADP(+))	**0.00E+ 00**	0.00E+ 00	**0.00E+ 00**	0.00E+ 00	**5.82E-02**	5.82E-02
Inositol 2-dehydrogenase	**6.00E-03**	6.00E-03	**5.00E-03**	5.00E-03	**6.40E-01**	5.56E-01
Mannitol-1-phosphate 5-dehydrogenase	**9.98E-02**	5.39E-02	**1.14E-01**	6.64E-02	**4.39E-01**	1.24E-01
Morphine 6-dehydrogenase	**0.00E+ 00**	0.00E+ 00	**3.42E-02**	3.42E-02	**5.03E-01**	4.17E-01
2,5-didehydrogluconate reductase (2-dehydro-D-gluconate-forming)^bc^	**1.01E-01**	3.93E-02	**1.58E-01**	8.51E-02	**2.31E+ 00**	9.38E-01
Serine 3-dehydrogenase (NADP(+))^c^	**0.00E+ 00**	0.00E+ 00	**0.00E+ 00**	0.00E+ 00	**1.22E-01**	4.33E-02
Aryl-alcohol dehydrogenase (NADP(+))	**0.00E+ 00**	0.00E+ 00	**0.00E+ 00**	0.00E+ 00	**3.10E-01**	2.01E-01
Glycerol-3-phosphate dehydrogenase (NAD(P)(+))^c^	**4.07E+ 00**	1.12E+ 00	**2.76E+ 00**	5.49E-01	**5.24E+ 00**	1.88E+ 00
Choline dehydrogenase^c^	**3.50E-03**	2.18E-03	**1.92E-02**	9.13E-03	**1.08E-01**	3.16E-02
Phenylalanine 4-monooxygenase	**0.00E+ 00**	0.00E+ 00	**0.00E+ 00**	0.00E+ 00	**1.18E-01**	9.24E-02
Ferroxidase	**8.87E-01**	3.36E-01	**1.17E+ 00**	6.54E-01	**1.78E+ 00**	4.83E-01
Cob(II)yrinic acid a,c-diamide reductase	**0.00E+ 00**	0.00E+ 00	**0.00E+ 00**	0.00E+ 00	**4.96E-02**	4.62E-02
Bacterial non-heme ferritin^c^	**1.06E+ 00**	5.80E-01	**5.15E-01**	1.48E-01	**3.52E+ 00**	9.58E-01
4-hydroxy-tetrahydrodipicolinate reductase	**6.22E+ 00**	1.99E+ 00	**6.63E+ 00**	1.67E+ 00	**5.67E+ 00**	1.58E+ 00
Succinylglutamate-semialdehyde dehydrogenase	**5.50E-03**	5.50E-03	**4.40E-03**	4.40E-03	**1.03E-01**	4.80E-02
3-methyl-2-oxobutanoate dehydrogenase (2-methylpropanoyl-transferring)	**0.00E+ 00**	0.00E+ 00	**0.00E+ 00**	0.00E+ 00	**5.10E-02**	2.46E-02
3-methyl-2-oxobutanoate dehydrogenase (ferredoxin)	**0.00E+ 00**	0.00E+ 00	**0.00E+ 00**	0.00E+ 00	**7.68E-01**	4.19E-01
D-proline reductase (dithiol)^c^	**6.95E-02**	6.95E-02	**0.00E+ 00**	0.00E+ 00	**4.38E-01**	1.76E-01
Betaine reductase^bc^	**0.00E+ 00**	0.00E+ 00	**0.00E+ 00**	0.00E+ 00	**1.06E-01**	4.84E-02
Trans-2-enoyl-CoA reductase (NAD(+))^b^	**0.00E+ 00**	0.00E+ 00	**0.00E+ 00**	0.00E+ 00	**1.57E-01**	1.01E-01
Dihydroorotate dehydrogenase (quinone)^c^	**2.70E-01**	1.03E-01	**2.87E-01**	9.68E-02	**9.19E-01**	2.00E-01
Glycine dehydrogenase (aminomethyl-transferring)^c^	**1.22E-01**	7.75E-02	**4.20E-02**	2.58E-02	**1.40E+ 00**	8.34E-01
Methylenetetrahydrofolate reductase (NAD(P)H)*^ab^*	**7.13E+ 00**	8.30E-01	**3.00E+ 00**	7.71E-01	**2.64E+ 00**	6.15E-01
FMN reductase (NADH)	**0.00E+ 00**	0.00E+ 00	**0.00E+ 00**	0.00E+ 00	**2.04E-02**	1.05E-02
Carboxynorspermidine synthase	**0.00E+ 00**	0.00E+ 00	**0.00E+ 00**	0.00E+ 00	**6.36E-02**	4.61E-02
Nitrite reductase (NAD(P)H)	**4.23E-02**	2.19E-02	**1.40E-01**	4.41E-02	**2.05E-01**	1.07E-01
Peptide-methionine (R)-S-oxide reductase^c^	**5.60E-01**	3.05E-01	**1.28E-01**	4.47E-02	**8.28E-01**	2.19E-01
Phosphatidylethanolamine N-methyltransferase^c^	**8.75E-03**	6.13E-03	**0.00E+ 00**	0.00E+ 00	**2.38E-01**	1.44E-01
16S rRNA (guanine(1207)-N(2))-methyltransferase^bc^	**8.75E-03**	8.75E-03	**1.28E-02**	1.11E-02	**2.18E-01**	1.16E-01
16S rRNA (cytosine(967)-C(5))-methyltransferase^bc^	**7.50E-04**	7.50E-04	**1.00E-03**	1.00E-03	**4.40E-02**	2.47E-02
16S rRNA (cytosine(1407)-C(5))-methyltransferase	**0.00E+ 00**	0.00E+ 00	**3.60E-03**	2.20E-03	**4.74E-02**	3.23E-02
23S rRNA (adenine(2085)-N(6))-dimethyltransferase	**7.30E-02**	5.02E-02	**3.12E-01**	2.91E-01	**1.16E-02**	7.28E-03
23S rRNA (uracil(747)-C(5))-methyltransferase^bc^	**5.25E-03**	5.25E-03	**1.80E-03**	1.80E-03	**2.17E-01**	9.84E-02
23S rRNA (cytosine(1962)-C(5))-methyltransferase^c^	**0.00E+ 00**	0.00E+ 00	**0.00E+ 00**	0.00E+ 00	**6.22E-02**	3.14E-02
Malonyl-[acyl-carrier protein] O-methyltransferase	**2.75E-03**	2.75E-03	**0.00E+ 00**	0.00E+ 00	**3.82E-02**	1.85E-02
16S rRNA (cytidine(1402)-2’-O)-methyltransferase	**2.66E+ 00**	4.41E-01	**2.11E+ 00**	5.15E-01	**3.81E+ 00**	1.59E+ 00
tRNA(1)(Val) (adenine(37)-N(6))-methyltransferase	**4.00E-03**	4.00E-03	**3.20E-03**	3.20E-03	**1.18E+ 00**	5.76E-01
Thymidylate synthase	**0.00E+ 00**	0.00E+ 00	**1.32E-02**	1.32E-02	**3.64E-01**	1.59E-01
3-methyl-2-oxobutanoate hydroxymethyltransferase^c^	**3.60E-01**	1.80E-01	**4.34E-01**	1.67E-01	**1.68E+ 00**	4.44E-01
Diaminobutyrate acetyltransferase^c^	**0.00E+ 00**	0.00E+ 00	**0.00E+ 00**	0.00E+ 00	**8.44E-02**	4.57E-02
Phosphinothricin acetyltransferase	**5.75E-03**	5.75E-03	**0.00E+ 00**	0.00E+ 00	**3.03E-01**	1.44E-01
Sulfoacetaldehyde acetyltransferase	**0.00E+ 00**	0.00E+ 00	**0.00E+ 00**	0.00E+ 00	**8.12E-02**	4.64E-02
Levansucrase^b^	**6.25E-03**	5.01E-03	**2.33E-01**	1.72E-01	**6.03E-01**	2.69E-01
4-O-beta-D-mannosyl-D-glucose phosphorylase^c^	**0.00E+ 00**	0.00E+ 00	**0.00E+ 00**	0.00E+ 00	**4.97E-01**	2.43E-01
Phosphatidylinositol alpha-mannosyltransferase^b^	**5.75E-03**	1.97E-03	**7.58E-02**	5.23E-02	**3.48E-01**	1.34E-01
Uridine phosphorylase^bc^	**1.10E-01**	4.26E-02	**8.52E-02**	5.84E-02	**5.43E-01**	2.14E-01
Triphosphoribosyl-dephospho-CoA synthase^c^	**1.73E-01**	8.60E-02	**5.94E-02**	1.82E-02	**2.22E-01**	4.83E-02
Diaminobutyrate – 2-oxoglutarate transaminase^c^	**0.00E+ 00**	0.00E+ 00	**0.00E+ 00**	0.00E+ 00	**5.72E-02**	3.89E-02
LL-diaminopimelate aminotransferase^bc^	**2.25E-03**	2.25E-03	**4.80E-03**	3.34E-03	**3.25E-01**	1.18E-01
Ribulokinase^bc^	**2.50E-03**	2.50E-03	**2.20E-03**	1.74E-03	**2.24E-01**	1.33E-01
Xylulokinase^c^	**1.13E-02**	7.18E-03	**9.20E-03**	8.01E-03	**8.02E-02**	4.34E-02
FAD:protein FMN transferase^c^	**4.40E-02**	2.46E-02	**7.80E-03**	7.80E-03	**1.25E-01**	3.97E-02
3-deoxy-D-manno-octulosonic acid kinase	**9.25E-03**	9.25E-03	**6.40E-03**	6.40E-03	**2.45E-01**	1.10E-01
Glycerol kinase^b^	**1.61E+ 00**	2.19E-01	**2.54E+ 00**	1.08E+ 00	**5.70E+ 00**	1.02E+ 00
Choline kinase^bc^	**6.61E+ 00**	6.34E+ 00	**3.57E-01**	7.27E-02	**2.98E-02**	1.86E-02
N-acetylglucosamine kinase^c^	**2.35E-02**	1.07E-02	**1.14E-02**	9.53E-03	**5.14E-01**	3.08E-01
Polyphosphate – glucose phosphotransferase^ab^	**4.08E-02**	2.09E-02	**6.76E-01**	5.17E-01	**1.05E+ 00**	5.30E-01
Inosine kinase^c^	**0.00E+ 00**	0.00E+ 00	**0.00E+ 00**	0.00E+ 00	**2.46E-01**	1.36E-01
Glucosamine kinase	**0.00E+ 00**	0.00E+ 00	**0.00E+ 00**	0.00E+ 00	**7.32E-02**	5.44E-02
Acetylglutamate kinase^bc^	**8.51E-01**	2.82E-01	**6.37E-01**	2.22E-01	**2.50E+ 00**	3.84E-01
[Glutamate – ammonia-ligase] adenylyltransferase^b^	**1.23E-01**	5.67E-02	**9.02E-02**	2.82E-02	**1.40E+ 00**	7.54E-01
Adenosylcobinamide-phosphate guanylyltransferase^c^	**4.25E-03**	4.25E-03	**0.00E+ 00**	0.00E+ 00	**9.92E-02**	5.32E-02
Diguanylate cyclase	**2.48E-02**	7.32E-03	**1.12E-02**	1.12E-02	**6.58E-02**	2.51E-02
Threonylcarbamoyladenylate synthase^c^	**4.96E-01**	1.39E-01	**3.61E-01**	1.12E-01	**8.56E-01**	3.43E-01
L-seryl-tRNA(Sec) selenium transferase	**4.75E-02**	1.60E-02	**2.88E-02**	1.68E-02	**4.28E-01**	1.30E-01
Protein-glutamate methylesterase^bc^	**0.00E+ 00**	0.00E+ 00	**9.40E-03**	7.98E-03	**4.64E-01**	2.16E-01
Nitrite reductase (NAD(P)H)	**4.23E-02**	2.19E-02	**1.40E-01**	4.41E-02	**2.05E-01**	1.07E-01
Peptide-methionine (R)-S-oxide reductase^c^	**5.60E-01**	3.05E-01	**1.28E-01**	4.47E-02	**8.28E-01**	2.19E-01
Phosphatidylethanolamine N-methyltransferase	**8.75E-03**	6.13E-03	**0.00E+ 00**	0.00E+ 00	**2.38E-01**	1.44E-01
16S rRNA (guanine(1207)-N(2))-methyltransferase	**8.75E-03**	8.75E-03	**1.28E-02**	1.11E-02	**2.18E-01**	1.16E-01
16S rRNA (cytosine(967)-C(5))-methyltransferase	**7.50E-04**	7.50E-04	**1.00E-03**	1.00E-03	**4.40E-02**	2.47E-02
16S rRNA (cytosine(1407)-C(5))-methyltransferase	**0.00E+ 00**	0.00E+ 00	**3.60E-03**	2.20E-03	**4.74E-02**	3.23E-02
23S rRNA (adenine(2085)-N(6))-dimethyltransferase	**7.30E-02**	5.02E-02	**3.12E-01**	2.91E-01	**1.16E-02**	7.28E-03
23S rRNA (uracil(747)-C(5))-methyltransferase	**5.25E-03**	5.25E-03	**1.80E-03**	1.80E-03	**2.17E-01**	9.84E-02
23S rRNA (cytosine(1962)-C(5))-methyltransferase	**0.00E+ 00**	0.00E+ 00	**0.00E+ 00**	0.00E+ 00	**6.22E-02**	3.14E-02
Malonyl-[acyl-carrier protein] O-methyltransferase	**2.75E-03**	2.75E-03	**0.00E+ 00**	0.00E+ 00	**3.82E-02**	1.85E-02
16S rRNA (cytidine(1402)-2’-O)-methyltransferase	**2.66E+ 00**	4.41E-01	**2.11E+ 00**	5.15E-01	**3.81E+ 00**	1.59E+ 00
tRNA(1)(Val) (adenine(37)-N(6))-methyltransferase	**4.00E-03**	4.00E-03	**3.20E-03**	3.20E-03	**1.18E+ 00**	5.76E-01
Thymidylate synthase	**0.00E+ 00**	0.00E+ 00	**1.32E-02**	1.32E-02	**3.64E-01**	1.59E-01
3-methyl-2-oxobutanoate hydroxymethyltransferase	**3.60E-01**	1.80E-01	**4.34E-01**	1.67E-01	**1.68E+ 00**	4.44E-01
Diaminobutyrate acetyltransferase	**0.00E+ 00**	0.00E+ 00	**0.00E+ 00**	0.00E+ 00	**8.44E-02**	4.57E-02
Phosphinothricin acetyltransferase	**5.75E-03**	5.75E-03	**0.00E+ 00**	0.00E+ 00	**3.03E-01**	1.44E-01
Sulfoacetaldehyde acetyltransferase	**0.00E+ 00**	0.00E+ 00	**0.00E+ 00**	0.00E+ 00	**8.12E-02**	4.64E-02
Levansucrase	**6.25E-03**	5.01E-03	**2.33E-01**	1.72E-01	**6.03E-01**	2.69E-01
4-O-beta-D-mannosyl-D-glucose phosphorylase	**0.00E+ 00**	0.00E+ 00	**0.00E+ 00**	0.00E+ 00	**4.97E-01**	2.43E-01
Phosphatidylinositol alpha-mannosyltransferase	**5.75E-03**	1.97E-03	**7.58E-02**	5.23E-02	**3.48E-01**	1.34E-01
Uridine phosphorylase	**1.10E-01**	4.26E-02	**8.52E-02**	5.84E-02	**5.43E-01**	2.14E-01
Triphosphoribosyl-dephospho-CoA synthase	**1.73E-01**	8.60E-02	**5.94E-02**	1.82E-02	**2.22E-01**	4.83E-02
Diaminobutyrate – 2-oxoglutarate transaminase	**0.00E+ 00**	0.00E+ 00	**0.00E+ 00**	0.00E+ 00	**5.72E-02**	3.89E-02
LL-diaminopimelate aminotransferase	**2.25E-03**	2.25E-03	**4.80E-03**	3.34E-03	**3.25E-01**	1.18E-01
Ribulokinase	**2.50E-03**	2.50E-03	**2.20E-03**	1.74E-03	**2.24E-01**	1.33E-01
Xylulokinase	**1.13E-02**	7.18E-03	**9.20E-03**	8.01E-03	**8.02E-02**	4.34E-02
FAD:protein FMN transferase	**4.40E-02**	2.46E-02	**7.80E-03**	7.80E-03	**1.25E-01**	3.97E-02
3-deoxy-D-manno-octulosonic acid kinase	**9.25E-03**	9.25E-03	**6.40E-03**	6.40E-03	**2.45E-01**	1.10E-01
Glycerol kinase	**1.61E+ 00**	2.19E-01	**2.54E+ 00**	1.08E+ 00	**5.70E+ 00**	1.02E+ 00
Choline kinase	**6.61E+ 00**	6.34E+ 00	**3.57E-01**	7.27E-02	**2.98E-02**	1.86E-02
N-acetylglucosamine kinase	**2.35E-02**	1.07E-02	**1.14E-02**	9.53E-03	**5.14E-01**	3.08E-01
Polyphosphate – glucose phosphotransferase	**4.08E-02**	2.09E-02	**6.76E-01**	5.17E-01	**1.05E+ 00**	5.30E-01
Inosine kinase	**0.00E+ 00**	0.00E+ 00	**0.00E+ 00**	0.00E+ 00	**2.46E-01**	1.36E-01
Glucosamine kinase	**0.00E+ 00**	0.00E+ 00	**0.00E+ 00**	0.00E+ 00	**7.32E-02**	5.44E-02
Acetylglutamate kinase	**8.51E-01**	2.82E-01	**6.37E-01**	2.22E-01	**2.50E+ 00**	3.84E-01
[Glutamate – ammonia-ligase] adenylyltransferase	**1.23E-01**	5.67E-02	**9.02E-02**	2.82E-02	**1.40E+ 00**	7.54E-01
Adenosylcobinamide-phosphate guanylyltransferase	**4.25E-03**	4.25E-03	**0.00E+ 00**	0.00E+ 00	**9.92E-02**	5.32E-02
Diguanylate cyclase	**2.48E-02**	7.32E-03	**1.12E-02**	1.12E-02	**6.58E-02**	2.51E-02
Threonylcarbamoyladenylate synthase	**4.96E-01**	1.39E-01	**3.61E-01**	1.12E-01	**8.56E-01**	3.43E-01
Alkaline phosphatase	**0.00E+ 00**	0.00E+ 00	**0.00E+ 00**	0.00E+ 00	**3.11E-01**	1.41E-01
2’,3’-cyclic-nucleotide 2’-phosphodiesterase	**1.63E-01**	6.62E-02	**1.81E-01**	7.27E-02	**3.83E-01**	2.92E-01
Beta-glucuronidase^b^	**1.13E-02**	6.42E-03	**3.26E-02**	1.59E-02	**1.01E-01**	2.61E-02
Beta-N-acetylhexosaminidase	**1.56E+ 00**	4.68E-01	**1.80E+ 00**	4.68E-01	**3.12E+ 00**	1.06E+ 00
Levanase^c^	**0.00E+ 00**	0.00E+ 00	**0.00E+ 00**	0.00E+ 00	**2.13E-01**	1.08E-01
Arabinogalactan endo-beta-1,4-galactanase	**0.00E+ 00**	0.00E+ 00	**0.00E+ 00**	0.00E+ 00	**6.43E-01**	3.24E-01
Glucan 1,6-alpha-isomaltosidase	**0.00E+ 00**	0.00E+ 00	**0.00E+ 00**	0.00E+ 00	**4.11E-01**	2.06E-01
Uracil-DNA glycosylase^c^	**2.16E+ 00**	9.31E-01	**1.21E+ 00**	2.87E-01	**4.62E+ 00**	1.52E+ 00
PepB aminopeptidase^bc^	**2.80E-02**	2.24E-02	**1.14E-02**	6.23E-03	**3.02E-01**	9.27E-02
Gamma-D-glutamyl-meso-diaminopimelate peptidase^c^	**8.78E-02**	5.96E-02	**1.32E-01**	7.75E-02	**0.00E+ 00**	0.00E+ 00
Prolyl oligopeptidase	**0.00E+ 00**	0.00E+ 00	**0.00E+ 00**	0.00E+ 00	**2.68E-02**	1.64E-02
Repressor LexA^c^	**1.22E+ 00**	9.76E-02	**1.07E+ 00**	4.27E-01	**4.25E+ 00**	1.38E+ 00
Gingipain R	**0.00E+ 00**	0.00E+ 00	**0.00E+ 00**	0.00E+ 00	**1.54E-01**	1.10E-01
Microbial collagenase	**0.00E+ 00**	0.00E+ 00	**0.00E+ 00**	0.00E+ 00	**6.62E-02**	3.70E-02
Mycothiol S-conjugate amidase^b^	**3.25E-02**	1.63E-02	**1.22E-01**	4.89E-02	**3.00E-01**	1.23E-01
Urease^bc^	**2.35E-01**	1.19E-01	**3.57E-01**	8.34E-02	**3.39E+ 00**	1.07E+ 00
Arginine deiminase	**3.78E+ 00**	1.51E+ 00	**3.02E+ 00**	1.27E+ 00	**8.11E+ 00**	3.76E+ 00
Adenosine deaminase^bc^	**1.69E-01**	4.30E-02	**3.15E-01**	8.53E-02	**8.10E-01**	2.08E-01
NAD(+) diphosphatase^c^	**1.03E-02**	4.87E-03	**0.00E+ 00**	0.00E+ 00	**2.36E-01**	1.16E-01
Guanosine-5’-triphosphate,3’-diphosphate diphosphatase^c^	**7.75E-03**	4.48E-03	**8.00E-03**	8.00E-03	**1.61E-01**	5.63E-02
Glycerol-3-phosphate-transporting ATPase	**0.00E+ 00**	0.00E+ 00	**2.60E-03**	2.60E-03	**1.92E-01**	1.65E-01
Cu(+) exporting ATPase^c^	**0.00E+ 00**	0.00E+ 00	**0.00E+ 00**	0.00E+ 00	**5.46E-02**	3.30E-02
b Phosphoribosylaminoimidazole carboxylase^b^	**2.75E-02**	1.05E-02	**7.18E-02**	2.72E-02	**1.74E-01**	2.98E-02
Phosphoenolpyruvate carboxykinase (GTP)^c^	**3.11E-01**	1.48E-01	**4.43E-01**	1.67E-01	**2.22E+ 00**	4.87E-01
2-oxoglutarate decarboxylase^bc^	**3.90E-02**	2.54E-02	**4.94E-02**	2.79E-02	**3.64E-01**	8.45E-02
3-dehydro-L-gulonate-6-phosphate decarboxylase	**6.25E-03**	6.25E-03	**2.20E-03**	2.20E-03	**1.51E-01**	7.97E-02
Aminodeoxychorismate lyase	**0.00E+ 00**	0.00E+ 00	**6.20E-03**	4.32E-03	**1.00E-01**	4.01E-02
Tryptophanase^bc^	**9.55E-02**	3.74E-02	**6.14E-02**	1.92E-02	**8.42E-01**	3.11E-01
Ectoine synthase	**0.00E+ 00**	0.00E+ 00	**0.00E+ 00**	0.00E+ 00	**8.40E-02**	6.57E-02
L(+)-tartrate dehydratase^c^	**1.60E-01**	1.34E-01	**7.16E-02**	2.46E-02	**9.19E-01**	3.71E-01
Myo-inosose-2 dehydratase	**0.00E+ 00**	0.00E+ 00	**5.00E-03**	5.00E-03	**6.22E-01**	3.49E-01
GDP-mannose 4,6-dehydratase^c^	**3.00E-01**	2.88E-01	**3.74E-02**	3.06E-02	**1.13E+ 00**	4.85E-01
1-aminocyclopropane-1-carboxylate synthase	**0.00E+ 00**	0.00E+ 00	**0.00E+ 00**	0.00E+ 00	**6.60E-02**	6.60E-02
Cellobiose epimerase	**0.00E+ 00**	0.00E+ 00	**0.00E+ 00**	0.00E+ 00	**4.85E-01**	2.25E-01
N-acylglucosamine-6-phosphate 2-epimerase	**2.40E+ 00**	1.33E+ 00	**1.87E+ 00**	8.83E-01	**1.99E+ 00**	9.10E-01
L-rhamnose isomerase^c^	**6.23E-02**	3.44E-02	**1.82E-02**	7.00E-03	**1.28E+ 00**	6.14E-01
Xylose isomerase^bc^	**2.38E-02**	8.63E-03	**5.86E-02**	4.50E-02	**1.08E+ 00**	6.12E-01
23S rRNA pseudouridine(2457) synthase	**0.00E+ 00**	0.00E+ 00	**0.00E+ 00**	0.00E+ 00	**1.52E-01**	1.31E-01
23S rRNA pseudouridine (955/2504/2580) synthase	**0.00E+ 00**	0.00E+ 00	**0.00E+ 00**	0.00E+ 00	**7.30E-02**	4.22E-02
RNA pseudouridine(65) synthase	**0.00E+ 00**	0.00E+ 00	**0.00E+ 00**	0.00E+ 00	**8.66E-02**	6.99E-02
RNA pseudouridine(32) synthase^bc^	**3.25E-03**	3.25E-03	**2.20E-03**	2.20E-03	**1.69E-01**	9.64E-02
23S rRNA pseudouridine (746) synthase^bc^	**3.25E-03**	3.25E-03	**2.20E-03**	2.20E-03	**1.69E-01**	9.64E-02
Acetate – CoA ligase	**0.00E+ 00**	0.00E+ 00	**1.47E-01**	1.47E-01	**3.80E-02**	2.47E-02
Long-chain-fatty-acid – CoA ligase^bc^	**1.57E-01**	7.10E-02	**1.46E-01**	6.37E-02	**6.88E-01**	1.93E-01
Asparagine synthase (glutamine-hydrolyzing)	**0.00E+ 00**	0.00E+ 00	**5.40E-03**	4.47E-03	**2.07E-01**	1.07E-01

P ≤ 0.05 a = CF vs CC, b = CF vs DC, c = CC vs DC
